# Identification of quantitative trait loci for dynamic and steady-state photosynthetic traits in a barley mapping population

**DOI:** 10.1093/aobpla/plaa063

**Published:** 2020-11-24

**Authors:** William T Salter, Si Li, Peter M Dracatos, Margaret M Barbour

**Affiliations:** 1 School of Life and Environmental Sciences, Sydney Institute of Agriculture, The University of Sydney, Brownlow Hill, NSW, Australia; 2 Plant Breeding Institute, The University of Sydney, Cobbitty, NSW, Australia; 3 School of Science, University of Waikato, Hillcrest, Hamilton, New Zealand

**Keywords:** Barley, dynamic photosynthesis, genotyping, mapping, phenotyping, Rubisco activation, sunfleck

## Abstract

Enhancing the photosynthetic induction response to fluctuating light has been suggested as a key target for improvement in crop breeding programmes, with the potential to substantially increase whole-canopy carbon assimilation and contribute to crop yield potential. Rubisco activation may be the main physiological process that will allow us to achieve such a goal. In this study, we assessed the phenotype of Rubisco activation rate in a doubled haploid (DH) barley mapping population [131 lines from a Yerong/Franklin (Y/F) cross] after a switch from moderate to saturating light. Rates of Rubisco activation were found to be highly variable across the mapping population, with a median activation rate of 0.1 min^−1^ in the slowest genotype and 0.74 min^−1^ in the fastest genotype. A unique quantitative trait locus (QTL) for Rubisco activation rate was identified on chromosome 7H. This is the first report on the identification of a QTL for Rubisco activation rate *in planta* and the discovery opens the door to marker-assisted breeding to improve whole-canopy photosynthesis of barley. This also suggests that genetic factors other than the previously characterized Rubisco activase (RCA) isoforms on chromosome 4H control Rubisco activity. Further strength is given to this finding as this QTL co-localized with QTLs identified for steady-state photosynthesis and stomatal conductance. Several other distinct QTLs were identified for these steady-state traits, with a common overlapping QTL on chromosome 2H, and distinct QTLs for photosynthesis and stomatal conductance identified on chromosomes 4H and 5H, respectively. Future work should aim to validate these QTLs under field conditions so that they can be used to aid plant breeding efforts.

## Teaser

Speeding up the photosynthetic induction response to fluctuating light has been highlighted as a key target for crop improvement programmes. We identified significant variation in the photosynthetic induction response after a switch from moderate to saturating light across a barley doubled haploid population. A quantitative trait locus (QTL) for Rubisco activation rate was identified on chromosome 7H, as well as overlapping QTLs for steady-state photosynthesis and stomatal conductance. This is the first report on the identification of a QTL for Rubisco activation rate *in planta* and the discovery opens the door to marker-assisted breeding to improve whole-canopy photosynthesis of barley.

## Introduction

By 2050, the global population is expected to rise to 9 billion and to meet future food demand we will need to increase crop production worldwide by 70 % (Paul *et al.* 2019). Recent progress has been hindered by stagnating rates of annual yield increase; therefore, novel breeding targets to improve crop yield potential are urgently needed. Improving the photosynthetic efficiency of crop species has now been shown to boost plant growth under field conditions (Kromdijk *et al.* 2016; South *et al.* 2019). Whilst these studies used genetic transformation to achieve such gains, they have proven that significant photosynthetic gains are possible in the field and that these can contribute to plant growth and crop yield. It is now imperative that we identify natural variation in photosynthetic traits in diverse populations and harness this variation through marker-assisted plant breeding techniques (Furbank *et al.* 2020).

Improving photosynthetic efficiency in dynamic environments has recently been highlighted as a key target to increase whole-canopy carbon assimilation (Murchie *et al.* 2018). The light environment of the lower canopy is subject to continuous and dynamic change across the course of a day, caused by movement of the sun across the sky, sporadic cloud cover and/or movement of upper elements in the canopy caused by wind (Slattery *et al.* 2018). These processes can cause a leaf in low/moderate light one moment to suddenly be exposed to saturating light conditions the next. Often these short periods of direct sunlit illumination (referred to herein as ‘sunflecks’) only last a short period of time, in the order of minutes, yet they can account for as much as 90 % of the daily accumulated light of lower canopy leaves (Pearcy 1990). Photosynthesis under these dynamic light conditions is highly inefficient. Specifically, the rates of stomatal opening and activation of Rubisco upon transition from low to high light significantly limit carbon assimilation. Interactive effects of several environmental factors on stomatal aperture are common in the field (Zeiger and Zhu 1998; Talbott *et al.* 2003; Wang *et al.* 2008), meaning that stomata are operating in an integrated and hierarchical manner in response to multiple environmental stimuli (Lawson and Blatt 2014). Stomatal responses to fluctuating light are therefore considered to be a much more challenging target for improvement than the biochemical limitations to photosynthesis (for a comprehensive review of limitations to dynamic photosynthesis, see Kaiser *et al.* 2019).

Improving Rubisco activation rate could be the low-hanging fruit that allows plant breeders to boost whole-canopy photosynthesis with few associated costs, specifically in terms of water and nutrient use. This is critical for a future where global environmental change is predicted to leave agricultural systems exposed to more frequent and more extreme drought and heat events. It has been estimated that we could increase daily carbon gain by as much as 21 % in wheat if Rubisco activation was instantaneous (Taylor and Long 2017). Variation in Rubisco activation kinetics has now been observed in crop species including soybean (Soleh *et al.* 2017), rice (Acevedo-Siaca *et al.* 2020) and wheat (Salter *et al.* 2019), and work with other species has indicated specific molecular targets and pathways that could accelerate Rubisco activation speed, with a particular focus on Rubisco’s catalytic chaperone Rubisco activase (Rca) (in *Arabidopsis thaliana*, Mott *et al.* 1997; in *Nicotiana tabacum*, Hammond *et al.* 1998; and in *Oryza sativa*, Yamori *et al.* 2012). In our recent work with wheat, we found that by increasing the rate of Rubisco activation of the slowest genotype included in our study to that of the fastest, daily carbon assimilation could be increased by 3.4 % (Salter *et al.* 2019). However, in this work only 10 genotypes of wheat were studied, the potential for improvement would likely be far more substantial if we were to investigate variation in this trait across a whole breeding population, and greater still if we were to investigate diversity within cultivars from diverse geographic locations or landraces.

Most recent studies of photosynthetic induction have tended to adopt the so-called ‘dynamic *A*/*c*_i_’ method (Taylor and Long 2017; Salter *et al.* 2019), in which photosynthetic induction curves are measured at a number of different CO_2_ concentrations, allowing for the reconstruction of *A*/*c*_i_ curves throughout the induction response. Whilst this technique yields important fundamental data on the biochemical limitations during photosynthetic induction (most importantly Rubisco carboxylation capacity, *V*_cmax_; and potential electron transport rate, *J*), it takes a very long time (>6 h per plant) and thus limits its use in large-scale screenings for photosynthetic induction traits. Conversely, other techniques that yield less detailed information about the underlying physiology (such as that used by Soleh *et al.* 2016) take far less time (<1 h) and may be much more suitable for large-scale screenings of diverse plant material. The method of Soleh *et al.* (2016) involves measuring a single photosynthetic induction curve at a low intracellular CO_2_ concentration (i.e. <300 μmol mol), at which it can be assumed that photosynthetic biochemistry is limited by Rubisco rather than by electron transport. This allows for the reliable estimation of Rubisco activation rate, with data comparable to those obtained using the ‘dynamic *A*/*c*_i_’ method (Taylor and Long 2017).

We hypothesize that variation in photosynthetic induction kinetics may be inadvertently confounding efforts to improve steady-state properties of photosynthesis. Steady-state measurement techniques [such as spot measurements of photosynthesis (*A*) and stomatal conductance (*g*_s_), CO_2_ response curves and light response curves] all rely on the assumption that the leaf is fully acclimated to saturating light, and other environmental conditions inside the leaf chamber of the system, prior to measurement. Thus, these methods require a delay for the leaf to become equilibrated to the conditions inside the leaf chamber of the gas exchange system (referred to herein as the equilibration time). Although it is quite well established that adequate equilibration time is required for accuracy of steady-state gas exchange measurements, an increasing demand for faster, higher-throughput measurement techniques (Furbank and Tester 2011) may make researchers complacent. Yet, few studies have quantitatively assessed the potential implications that may result from premature assumptions of steady-state conditions, for instance, the identification of false quantitative trait loci (QTLs).

There is now compelling evidence that suggests whole-canopy photosynthesis could be improved by harnessing natural variation in Rubisco activation rate that exists across genotypes of crop species. However, no study to date has investigated or performed trait dissection for Rubisco activation in a segregating mapping population. We sought to identify and characterize genetic variation in Rubisco activation rate across a barley (*Hordeum vulgare*) doubled haploid (DH) mapping population *in planta* using gas exchange techniques. We then used chromosome interval mapping to identify QTLs and closely associated molecular markers. We were also interested in assessing whether the strength of QTLs would be weakened for ‘steady-state’ photosynthetic properties (*A* and *g*_s_) if equilibration times were not long enough for steady-state conditions to be reached.

## Methods

### Plant material and growth conditions

A DH barley (*H. vulgare*) population was obtained from a cross between the Australian barley cultivars Yerong and Franklin (Y/F). This population contained 177 DH lines and was maintained at the Plant Breeding Institute at The University of Sydney. The Y/F mapping population has been extensively used for QTL mapping for both morphological (Xue *et al.* 2008) and physiological (Zhang *et al.* 2016) traits, as well as disease resistance (Singh *et al.* 2015; Dracatos *et al.* 2016). In this study 131 lines from this population were phenotypically assessed for steady-state and dynamic photosynthetic traits. Due to the availability of seed and genotypic data, only 127 DH lines were used for QTL analyses. A second DH barley population (from a cross between VB9104 and Dash) was also phenotyped for photosynthetic traits; however, due to the low number of lines with available genotypic data this population was not included in further analyses (phenotyping results are however presented in **Supporting Information—Figs S4**  **and**  **S5**).

Plants were grown in a controlled environment room for ~5 weeks prior to measurement and were measured at the advanced tillering stage. Day temperature was 25 °C during a 14-h light period and night temperature was 17 °C during a 10-h dark period. Relative humidity was maintained at 70 % while daytime Photosynthetic photon flux density (PPFD) was ~600 μmol m^−2^ s^−1^ at the top of the plants. Seeds were planted in potting mix enriched with slow-release fertilizer (Osmocote Exact, Scotts, Sydney, NSW, Australia). Six seeds per genotype were sown in 6-L pots and grown for 3 weeks before being thinned to three plants per pot. Seed was sown sequentially in time to make sure that all measurements were conducted at the same growth stage. Plants were watered daily to field capacity.

### Photosynthetic measurements

Plants were moved from the controlled environment room to an adjacent temperature-controlled growth cabinet (TPG-2900-TH-3218; Thermoline Scientific, Wetherill Park, NSW, Australia) under the same environmental conditions (temperature 25 °C; relative humidity 70 %). Care was taken to minimize the length of time that the plants were in transit between the controlled environment room and the growth cabinet; however, we note that this transition was not instantaneous. Two or three of the youngest fully expanded leaves of a single plant were sealed in a 2 × 6 cm leaf cuvette (6400-11; LI-COR, Lincoln, NE, USA) fitted to a LI-COR LI-6400XT gas exchange system to fill the cuvette without overlapping. This simulated a shift in light intensity from 600 to 1300 μmol m^−2^ s^−1^. This moderate-to-high light transition allowed us to assess photosynthetic induction whilst overcoming complications often caused by low stomatal conductance of traditional dark-to-light transitions. Leaf chamber conditions were set to closely match those of the controlled environment room [leaf temperature 25 °C; cuvette CO_2_ (CO_2_S) 400 µmol mol^−1^; relative humidity 70 %], with the exception of PPFD which was set to 1300 µmol m^−2^ s^−1^ using a red–green–blue light source (6400-18A; LI-COR) set to 10 % blue and 90 % red light (set to replicate the commonly used settings of the standard 2 × 3 cm light source). Measurements of photosynthetic gas exchange rates (*A* and *g*_s_) were recorded once per minute immediately after the leaf was inserted into the chamber until photosynthesis had reached steady state. Logging was conducted at this frequency as the LI-COR system was also connected to a tuneable diode laser that required a low-logging frequency; however, if this study were to be repeated we would encourage logging more frequently. Preliminary photosynthetic light response curves were measured with plants grown under the same conditions to ensure that 1300 μmol m^−2^ s^−1^ was saturating and that 600 μmol m^−2^ s^−1^ was non-saturating (results shown in **Supporting Information—Fig. S1**).

**Figure 1.**
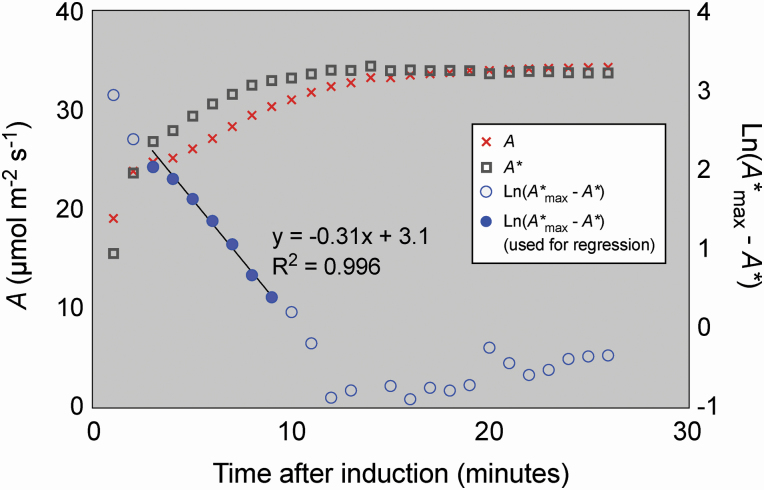
Example of a typical leaf photosynthetic induction response and the linear regression used to calculate Rubisco activation rate (1/τ). The orange crosses represent the measured photosynthetic rate *A*; the grey squares the *c*_i_ = 300 µmol mol^−1^ normalized photosynthetic rate A*; and the blue circles the logarithmic difference between the fully induced photosynthetic rate A* _max_ and A*. Filled circles represent the data points used in the linear regression to estimate 1/τ, the Rubisco activation rate. The slope of the regression represents 1/τ, in this case 0.31 min^−1^.

**Figure 2.**
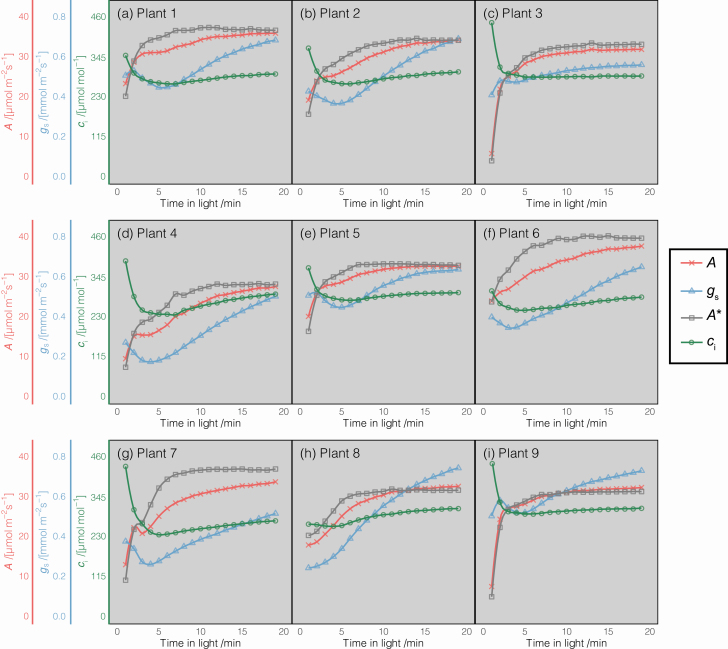
Induction curves for net photosynthesis, *A* (red crosses); stomatal conductance, *g*_s_ (blue triangles); *c*_i_ (green circles); and *c*_i_ = 300 ppm normalized photosynthesis, A* (grey squares), after a switch from moderate to saturating light. Data shown in panels (A)–(I) are representative induction curves from 1 day of measurements in individual plants. The value of 1/τ is shown in each panel for reference.

Rubisco activation rate was calculated using a modified method of Woodrow and Mott (1989) and Soleh *et al.* (2016). Photosynthetic data were first normalized to an intercellular CO_2_ concentration (*c*_i_) of 300 µmol mol^−1^, assuming that the relationship between *A* and *c*_i_ at any point in induction was described by a straight line through zero, using the following equation:

$$\begin{array}{l} A^{*}=A\;\times \frac{300}{c_{i}} \end{array}$$

where A* is the normalized photosynthetic rate, *A* is the measured photosynthetic rate and *c*_i_ is the measured intercellular CO_2_ concentration. This effectively removed the influence of stomatal opening/closure for the induction phase. The choice to correct to a *c*_i_ of 300 µmol mol^−1^ followed that of Soleh *et al*. but was also found to be close to the steady-state *c*_i_ of our plants (as shown in Fig. 2). We note that this choice did not affect subsequent calculation of the kinetics of Rubisco activation. The apparent Rubisco activation rate (1/τ) was modelled from the plot of the logarithmic difference between A* and its maximum value after induction (A* _max_) against the time taken for induction (representative data shown in Fig. 1). From this plot, the value of 1/τ was determined from the slope of the linear regression on data points in the range of 2–5 min after induction, and points after this that maintained a regression with *R*^2^ > 0.9. The first phase of induction (<2 min after transition to saturating light) was not included in the regression as the slope is not related to Rubisco activation before this point, with other processes such as increases in stromal pH having a strong influence on this early induction phase (Woodrow and Mott 1989). We refer to 1/τ as the apparent Rubisco activation rate here as we note that it could also be influenced by other photosynthetic processes, including dynamic responses of mesophyll conductance and non-photochemical quenching; however, in the remainder of the text we refer to it simply as Rubisco activation rate.

### Genetic analysis and QTL mapping

The genotypic data and genetic linkage map for the Y/F DH population used for QTL analysis for Rubisco activity and steady-state photosynthetic traits in the present study were previously described by Singh *et al*. (2015). In brief, the Y/F genetic map is comprised of 496 DarT and 28 microsatellite (Simple sequence repeat(s)) markers spanning 1127 cM across all seven chromosomes, 1H to 7H (Wenzl *et al.* 2006).

A subset of 127 lines were selected for QTL mapping analysis. Markers were selected every 10 cM so that the whole genome was evenly covered. Composite interval mapping (CIM) methods were used in QTL Cartographer version 2.5 (North Carolina State University, Raleigh, NC, USA), carrying out 1000 iterations permutation analysis with steps at 1 cM, and with a 0.05 confidence level for all traits.

### Statistical analyses

All other modelling and statistical analyses were performed in R (R Core Team 2019).

## Results

### Photosynthetic induction kinetics

While specific photosynthetic induction kinetics were found to vary across individual leaves and genotypes, general trends were quite clear (representative induction curves from 1 day of measurements are shown in Fig. 2). Net photosynthesis (*A*) increased immediately after transition from to saturating light for all leaves. Stomatal responses were more variable than those of photosynthesis but there tended to be an initial reduction in *g*_s_ after transition to saturating light and then a gradual rise towards steady state. By normalizing photosynthesis to a constant *c*_i_ of 300 ppm, we were able to obtain a measure of photosynthesis limited by Rubisco carboxylation unobstructed by variation in stomatal kinetics (A*). A* showed a similar trend to *A*, increasing immediately after the switch from low to high light.

### QTLs for Rubisco activation rate

**Figure 3.**
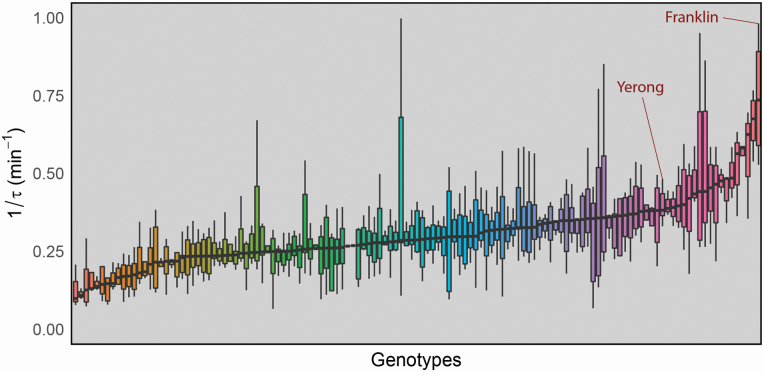
Distribution of Rubisco activation rate (1/τ) across genotypes of the Yerong/Franklin DH population. Each bar represents a single genotype. Parental lines are highlighted. Colours are arbitrary.

**Figure 4.**
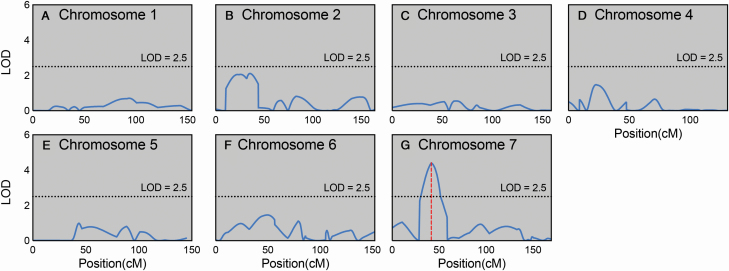
Logarithm of odds (LOD) traces from composite interval QTL mapping analysis for 1/τ. Logarithm of odds values are plotted against the position on the chromosomes. The significance threshold LOD of 2.5 is indicated by the dotted line in each plot. Vertical dashed red lines represent identified QTLs.

Rubisco activation rates of the parental lines Y/F were found to differ, with within-genotype medians of 0.38 and 0.74 min^−1^, respectively. Wide variation in 1/τ was found across the population (Fig. 3), with within-genotype medians ranging from 0.099 to 0.74 min^−1^. Interestingly, the parental line Franklin was found to have the fastest rate of Rubisco activation. A frequency distribution of 1/τ was plotted for the population and was found to follow a normal distribution suggesting that Rubisco activation rate was under complex genetic control **[see**  **Supporting Information—Fig. S2****]**. Composite interval mapping analysis revealed the presence of a distinct QTL for Rubisco activation rate (Fig. 4; further details in Table 1). Q1/τ.sun-7H was located at 41.67 cM on chromosome 7H (proximal to DarT marker bPb-9601) accounting for 10.48 % of the phenotypic variance in this trait.

**Table 1.** QTLs for dynamic and steady-state photosynthetic traits identified in the mapping population.

Trait	QTL	Chromosome	Position (cM)	Nearest marker	Explained variance (%)	Additivity	LOD
1/τ	Q1/τ.sun-7H	7H	41.67	bpb-9601	10.48	−0.07	4.40
*A*	Q*A*.sun-2H	2H	27.03	bpb-0003	9.20	−3.85	4.31
*A*	Q*A*.sun-4H	4H	66.68	bpb-2305	5.84	−4.37	2.64
*A*	Q*A*.sun-7H	7H	41.67	bpb-9601	10.80	−4.30	5.18
*g*_s_	Q*g*_s_.sun-2H	2H	35.91	bpb-8750	11.80	−0.09	5.41
*g*_s_	Q*g*_s_.sun-5H	5H	53.39	bpb-5532	6.49	0.07	2.98
*g*_s_	Q*g*_s_.sun-7H	7H	41.28	bpb-4989	13.75	−0.11	6.80

### Steady-state photosynthesis and equilibration time tests

**Figure 5.**
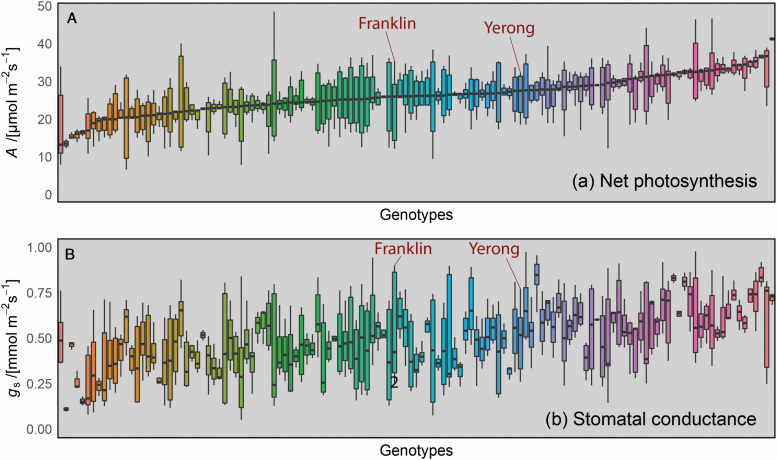
Distribution of steady-state (A) *A* and (B) *g*_s_ across genotypes of the Yerong/Franklin population. Each bar represents a single genotype. Parental lines are highlighted. Note that colours are arbitrary but are consistent for genotypes in panels (A) and (B).

**Figure 6.**
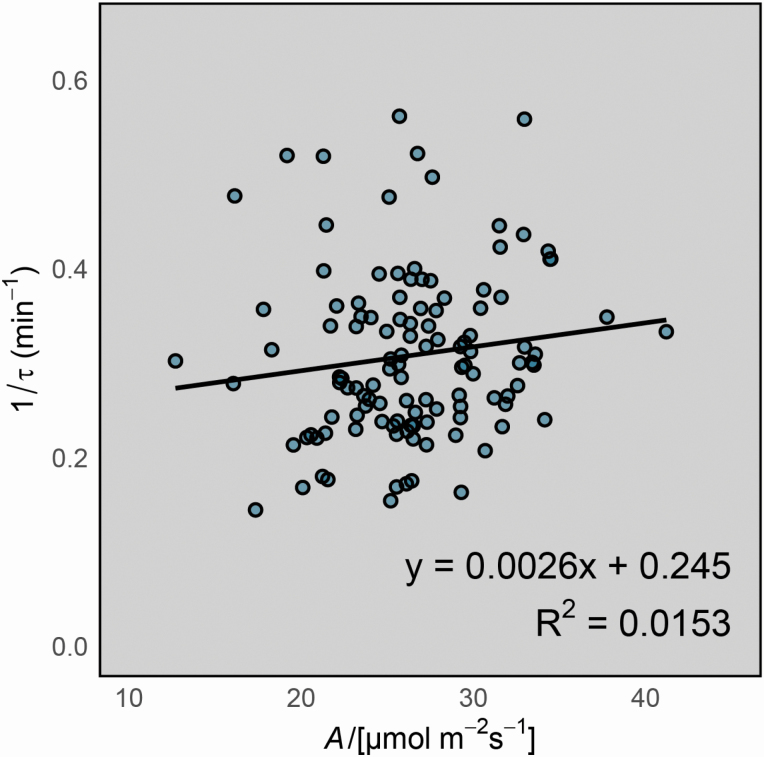
Relationship between steady-state *A* and 1/τ. Each point represents a genotype. Values are genotype means.

Variation was also found in steady-state photosynthetic rates across the population (Fig. 5). Median rates of *A* and *g*_s_ were 17.45 and 0.31 mmol m^−2^ s^−1^, respectively. From these phenotyping data, there was no correlation found between steady-state *A* and 1/τ (correlation coefficient = 0.0026; *P* > 0.05; Fig. 6).

As hypothesized, ‘steady-state’ photosynthetic rates were substantially underestimated if measurements were recorded without sufficient equilibration time (Table 2). This was more pronounced the earlier the measurements were recorded after enclosing the leaf in the chamber of the Infrared gas analyser (IRGA). Mean values of *A* and *g*_s_ were both underestimated by 21 % at 5 min compared to steady state. It should be noted that although some of the fastest genotypes reached steady state after 5 min, most of the lines did not. In fact, *g*_s_ was underestimated by 82 % for one of the genotypes and *A* was underestimated by 54 % for another if measurements were recorded after just 5 min.

**Table 2.** Distribution features for photosynthetic rate (*A*) and stomatal conductance (*g*_s_) across the population at 5, 10 and 15 min into photosynthetic induction.

Trait	Time point (minutes)	Minimum	25 % percentile	Median	75 % percentile	Maximum	Mean
*A* (µmol m^−2^ s^−1^)	5	7.1	16.5	20.0	24.9	36.5	20.4
	10	9.6	20.0	23.3	26.9	40.2	23.4
	15	12.2	21.5	24.9	28.4	41.8	25.0
*g*_s_ (mmol m^−2^ s^−1^)	5	0.044	0.259	0.365	0.522	0.947	0.397
	10	0.069	0.294	0.392	0.508	0.947	0.412
	15	0.107	0.330	0.441	0.544	0.940	0.451

To assess the importance of equilibration time for accurate identification of steady-state QTLs, QTL mapping was first performed for steady-state *A* and *g*_s_. Frequency distributions were plotted for both traits and they followed a normal distribution suggesting they are under complex genetic control **[see**  **Supporting Information—Fig. S3****]**. Several QTL were found for both *A* and *g*_s_ (Table 1). Trait co-location was observed on chromosome 7H whereby the position of the Q1/τ.sun-7H QTL was almost identical to QTL for both *A* and *g*_s_. This suggests a region on the short arm of chromosome 7H either carries a single gene or more likely a cluster of genes responsible for the genetic control of photosynthesis, stomatal conductance and Rubisco activation. For steady-state *A* and *g*_s_, additional overlapping and distinct QTL were identified. A common overlapping QTL for both *A* and *g*_s_ was identified, peaking at 27.03 cM on chromosome 2H, whilst distinct QTL were identified on chromosomes 4H (41.67 cM) and 5H (53.39 cM) for *A* and *g*_s_, respectively.

**Figure 7.**
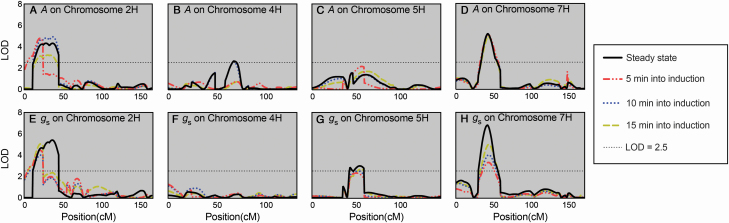
Logarithm of odds (LOD) traces from composite interval QTL mapping analysis for *A* and *g*_s_ in the Yerong/Franklin DH population. Logarithm of odds values are plotted against the cM position on chromosomes 2H, 4H, 5H and 7H. The threshold LOD of 2.5 is indicated by the horizontal dotted line in each plot. Note that LOD plots for chromosomes 1H, 3H and 6H are not shown as there were no significant QTLs identified on these chromosomes.

QTL mapping was then performed with data collected at 5, 10 and 15 min after the start of induction for comparison with detected steady-state QTLs (coloured traces in Fig. 7). Although most QTLs were still identified with non-steady-state data, the significance these QTL peaks were found to be weakened under non-steady-state conditions. This was particularly evident for the *g*_s_ QTL identified on chromosome 7H (Fig. 7H), with the LOD score of this QTL dropping from 6.8 when using steady-state data to 4.9, 3.9 and 3.3 when using data collected at 15, 10 and 5 min after the start of induction, respectively.

## Discussion

We have identified QTLs for *in planta* Rubisco activation rate for the first time in any species. As in other crops, we found Rubisco activation rate to be a highly variable trait across genotypes of barley, aiding in the discovery of a significant QTL in our DH population. Quantitative trait loci were also identified for steady-state photosynthetic parameters, including co-localized QTLs for *A*, *g*_s_ and 1/τ on chromosome 7H. The 7H QTL was distinct from the location of both Rubisco activase isoforms that map to the same region on chromosome 4H (Saghai Maroof *et al.* 1994), suggesting other genetic factors are also important for the regulation of Rubisco activation. The importance of adequate equilibration time in the measurement of steady-state gas exchange was highlighted by comparing these results to those obtained using arbitrary non-steady-state rates at 5, 10 and 15 min after the start of induction. The significance of QTLs was reduced if steady-state conditions had not been reached.

### Improving whole-canopy photosynthesis

It is well established that improving photosynthesis has the potential to increase crop yield (for a review of recent progress, see Simkin *et al.* 2019). However, until now research has invariably focussed on only the uppermost leaves of the canopy under optimal conditions (i.e. continuous saturating light, 25 °C). This approach has its merits because these leaves have the most light available to them and their contribution to whole-canopy photosynthesis reflects this (Osborne *et al*. 1998). Yet, for monocot cereal species such as wheat and barley there have been few studies that have shown flag leaf photosynthesis to correlate well with crop yield (Richards 2000). Whole-canopy photosynthesis, and more specifically the cumulative rate of photosynthesis over the growing season, has been shown through modelling studies to be a much more reliable determinant of crop yield (Wu *et al.* 2019). Accordingly, there has been a recent shift in research focus towards dynamic photosynthetic traits. This is important because whilst some studies have found weak relationships between steady-state and dynamic photosynthetic traits (Salter *et al.* 2019) other studies have not found any relationship (Soleh *et al.* 2017; Acevedo-Siaca *et al.* 2020). Our results also showed no link between steady-state *A* and 1/τ (Fig. 6), although the co-localization of QTLs on chromosome 7H suggests they both may be controlled by the action of a single gene or a cluster of closely linked genes at the same chromosomal location.

Significant improvements in photosynthesis and resultant increases in plant growth have now been achieved under field conditions through genetic modification of model plant species (15 % increased biomass production by accelerating recovery from photoprotection, Kromdijk *et al.* 2016; and 40 % increased biomass via engineering of a photorespiratory bypass, South *et al.* 2019) and recent modelling has highlighted the potential of improving several dynamic photosynthetic traits on whole-canopy photosynthesis (Wang *et al.* 2020). It is important now that we explore and exploit natural variation in photosynthetic traits across plant populations (for review, see Furbank *et al.* 2020). As in previous studies with other species, we identified significant variation in Rubisco activation rate across barley genotypes. We identified a QTL for Rubisco activation rate, as well as several QTLs for steady-state *A* and *g*_s_. Q1/τ.sun-7H was flanked by the bpb-9601 DArT marker which has previously been associated with both grain yield and crop spike number in the Y/F population (Xue *et al.* 2010). This marker is of particular interest as it also flanks QTLs that we identified for steady-state *A* and *g*_s_ (Q*A*.sun-7H and Q*g*_s_.sun-7H in Table 1), highlighting its utility for marker-assisted selection (MAS). Marker-assisted selection exploiting natural variation between barley genotypes can now be achieved through the development of a high-throughput codominant marker using the sequence information from the closely associated bpb-9601 DArT marker identified in this study. Marker-assisted selection for dynamic and steady-state photosynthetic traits in barley now provides potential to improve daily photosynthetic carbon gain in both sporadically sunlit lower canopy and fully sunlit upper canopy leaves, respectively, bolstering whole-canopy photosynthesis and contributing to yield potential.

Whilst we observed segregation for three different photosynthetic traits in the barley mapping population studied, and the V/D population presented in **Supporting Information—Figs S4**  **and**  **S5**, these populations were not specifically developed to investigate photosynthesis. Future work in this area would hugely benefit from phenotyping a diverse panel of barley accessions to either develop additional trait-specific mapping populations using parents with contrasting photosynthetic properties or use a genome-wide association scan (GWAS) approach to mine for novel favourable alleles based on natural variation in photosynthetic traits. This may also include the investigation of crop wild relatives (Castañeda-Álvarez *et al.* 2016). Such approaches have already yielded promising outcomes for other desirable traits in crop species, including salinity (in barley, Saade *et al.* 2016) and drought tolerance (Venuprasad *et al.* 2009).

Due to the recent availability of multiple reference genomes for cultivated and wild barley, the precision of GWAS studies and ability to rapidly clone genes of interest from cereal crops is continually improving. Further studies are required to determine whether each of the traits studied are under control by a single gene or more complex genetic control within the QTL region on chromosome 7H. Further mendelization of the 7H QTL by intercrossing select DH lines from the Y/F population will enable the development of a large segregating F_2_ fine-mapping population for positional cloning of the 7H QTL to unravel the underlying genetic and biological mechanisms involved.

Our study focussed on a step change from moderate (600 µmol m^−2^ s^−1^) to saturating light (1300 µmol m^−2^ s^−1^), rather than low to high light as has been reported previously (i.e. 50–1500 µmol m^−2^ s^−1^ in Taylor and Long 2017). We felt this approach would provide more valuable information for plant breeding, as it more accurately represents the light regime experienced by the second youngest leaves in the canopy, which for wheat have been reported to receive between 300 and 700 µmol m^−2^ s^−1^ PPFD when not in a sunfleck (Townsend *et al.* 2018). Whilst leaves lower in the canopy receive much less light than this (<300 µmol m^−2^ s^−1^), these leaves are also less likely to be exposed to sunflecks and also have a much-reduced photosynthetic capacity (Townsend *et al.* 2018), so contribute considerably less to whole-canopy photosynthesis. Our results show that Rubisco activation rates after a switch from moderate to high light in barley (median 1/τ = 0.28 min^−1^) are similar to those that have been reported from low to high light in other species (0.3–0.45 min^−1^ in rice, Yamori *et al.* 2012; 0.24–0.42 min^−1^ in soybean, Soleh *et al.* 2016; and 0.25–0.33 min^−1^ in wheat, Taylor and Long 2017), albeit with greater variation. It would therefore seem that the same biochemical processes, likely related to the amount of and form of Rubisco activase present in the leaves (Carmo-Silva and Salvucci 2013), are involved in photosynthetic induction under the two induction scenarios.

### Limitations and future directions

Our study has focussed on Rubisco activation; however, this is only one part of the dynamic photosynthesis puzzle, in which all the pieces must be investigated to fully understand potential improvements that could be made to whole-canopy photosynthesis. Responses of stomata can also limit photosynthesis in fluctuating light. Faster stomatal opening has now been shown to improve net photosynthesis and biomass production in overexpressing mutants of *A. thaliana* compared to wild type plants (Kimura *et al*. 2020). And so, if improvements are made to Rubisco activation rate without also considering rates of stomatal opening/closure, the dominant limitation will likely shift in the direction of the stomata. In effect, this could nullify any improvements made to Rubisco activation in terms of net photosynthesis. On a positive note, it has been shown that stomatal traits can be linked to Rubisco kinetics during leaf development in some plant species (Conesa *et al.* 2019), and it has long been realized that stomata respond to photosynthetic activity in the mesophyll (Messinger *et al.* 2006). Głowacka *et al*. (2018) recently confirmed the intimate link between photosynthetic biochemistry and stomata in field-grown crop plants. They showed that overexpression of *Photosystem II Subunit S* (*PsbS*), which directly affects the rate of energy absorption in photosystem II, results in reduced stomatal opening in response to light. Kromdijk *et al.* (2019) subsequently incorporated this link into a mechanistic model of photosynthesis and stomatal conductance. It is therefore conceivable that improving Rubisco activation rate through targeted plant breeding could also inherently result in improved stomatal responses. Regardless, there is a definite need for future work in this area to address dynamic responses of stomata, Rubisco and other biochemical processes (i.e. non-photochemical quenching) of photosynthesis together, rather than focussing on each in isolation.

In this study, we measured photosynthetic induction and identified associated QTLs in plants grown under optimal and controlled conditions. The next important step is for photosynthetic induction traits to be investigated in field-grown plants with established canopies. Traditional gas exchange techniques combined with new higher-throughput techniques based on thermography (for dynamic stomatal traits; Vialet-Chabrand and Lawson 2020), hyperspectral imaging and chlorophyll fluorescence (for dynamic photosynthetic parameters; McAusland *et al.* 2019; Meacham-Hensold *et al.* 2020) may offer the potential to screen these two populations in the field and validate the QTLs we identified in this study. It is also important that we understand if these QTLS are strong under suboptimal conditions (i.e. under drought or heat stress), as for most growers such conditions can be common during a growing season.

### A note on gas exchange methodology

It is common practice to allow a leaf to stabilize to the chamber conditions of an IRGA, yet the recent push for ‘high-throughput’ and ‘big data’ approaches in plant physiology may have made researchers complacent. We hypothesized that this complacency could impact detected QTLs for photosynthesis and stomatal conductance, and indeed we found that using non-steady-state rates (i.e. before leaves had equilibrated to chamber conditions) resulted in less accurate detection of QTLs. It is likely that false QTL identifications are worsened by the high variability in photosynthetic induction kinetics that exists across this population (and has also been found in other crop species) and the fact that there is no clear relationship between steady-state and dynamic photosynthesis. This result reinforces the importance of good gas exchange technique. The push for high-throughput measurements has resulted in new fast methods, such as the Rapid *A*/*c*_i_ method (Stinziano *et al.* 2017), being developed yet it must be highlighted that most of these methods still rely on the assumption of steady-state conditions and these will therefore still be limited by equilibration time.

We suggest that plant physiologists treat this as a methodological opportunity instead of a hindrance. Rather than just waiting for the leaf to reach steady state and then recording a point measurement or photosynthetic response curve, the photosynthetic induction phase could always be logged continuously as soon as the leaf enters the chamber. Not only would this provide extra data on photosynthetic induction, it would also give a researcher more confidence in their data. Specifically, they would be able to backcheck to ensure that steady-state conditions had been reached. In the past, technical limitations may have prevented such an approach, but new gas exchange instruments have the computational power, storage capacity and environmental control to establish this as common practice. New data analysis software, including the R packages {Plantecophys} (Duursma 2015) and {Photosynthesis} (Stinziano *et al.* 2020), that allow large gas exchange data sets to be quickly processed will further enable this change.

## Conclusions

In this study, we found wide variation in photosynthetic induction to fluctuating light across a barley mapping population. This variation allowed us to identify a QTL for Rubisco activation rate, the position of which overlapped QTLs for steady-state photosynthesis and stomatal conductance. These QTLs lie close to molecular markers that could be used for selection in plant breeding programmes. Future work should aim to validate these QTLs under field conditions so that they can be used to aid plant breeding efforts.

## Supporting Information

The following additional information is available in the online version of this article—

**Figure S1.** Photosynthetic light response curves measured on plants of the parental line Dash grown under the same growth conditions as experimental plants. Curves were fitted to a non-rectangular hyperbola model using non-linear least squares in R (*nls*; R Language and Environment) as per Salter *et al.* (2019). Vertical dashed lines are shown at 600 and 1300 μmol m^−2^ s^−1^ to highlight the moderate-to-high light induction phase measured in this study.

**Figure S2.** Frequency distributions of 1/τ for the (a) Y/F DH population.

**Figure S3.** Frequency distributions of steady-state (a) *A* and (b) *g*_s_ for the Y/F DH population.

**Figure S4.** Distribution of Rubisco activation rate (1/τ) across genotypes of the V/D DH population. Each bar represents a single genotype. Parental lines are highlighted. Colours are arbitrary.

**Figure S5.** Distribution of steady-state (a) *A* and (b) *g*_s_ across genotypes of the VB9104/Dash population. Each bar represents a single genotype. Parental lines are highlighted. Note that colours are arbitrary but are consistent for genotypes in panels (a) and (b).

plaa063_suppl_Supplementary_Figures

plaa063_suppl_Supplementary_File_S1

plaa063_suppl_Supplementary_File_S2

## Data Availability

The following files are available in the **Supporting Information**:

FileS1.xlsx – Yerong/Franklin dynamic and steady-state gas exchange phenotypic data.

FileS2.xlsx – Results of composite interval mapping of dynamic and steady-state photosynthetic traits.

## Notes

**Phenome, Genome & Environment. Chief Editor:** Colleen Doherty
