# Supplementary material for: Identification of quantitative trait loci for dynamic and steady-state photosynthetic traits in a barley mapping population
Source: AoB Plants. 2020 Nov 24;12(6):plaa063. doi: 10.1093/aobpla/plaa063 (PMC7759950; doi:10.1093/aobpla/plaa063)

Supplementary figures

Figure S1 – Photosynthetic light response curves measured on plants of the parental line Dash grown under the same growth conditions as experimental plants. Curves were fitted to a non-rectangular hyperbola model using non-linear least squares in R (*nls*; R Language and Environment) as per Salter *et al*. (2019). Vertical dashed lines are shown at 600 µmol m^{-2} s^{-1} and 1300 µmol m^{-2} s^{-1} to highlight the moderate to high light induction phase measured in this study.

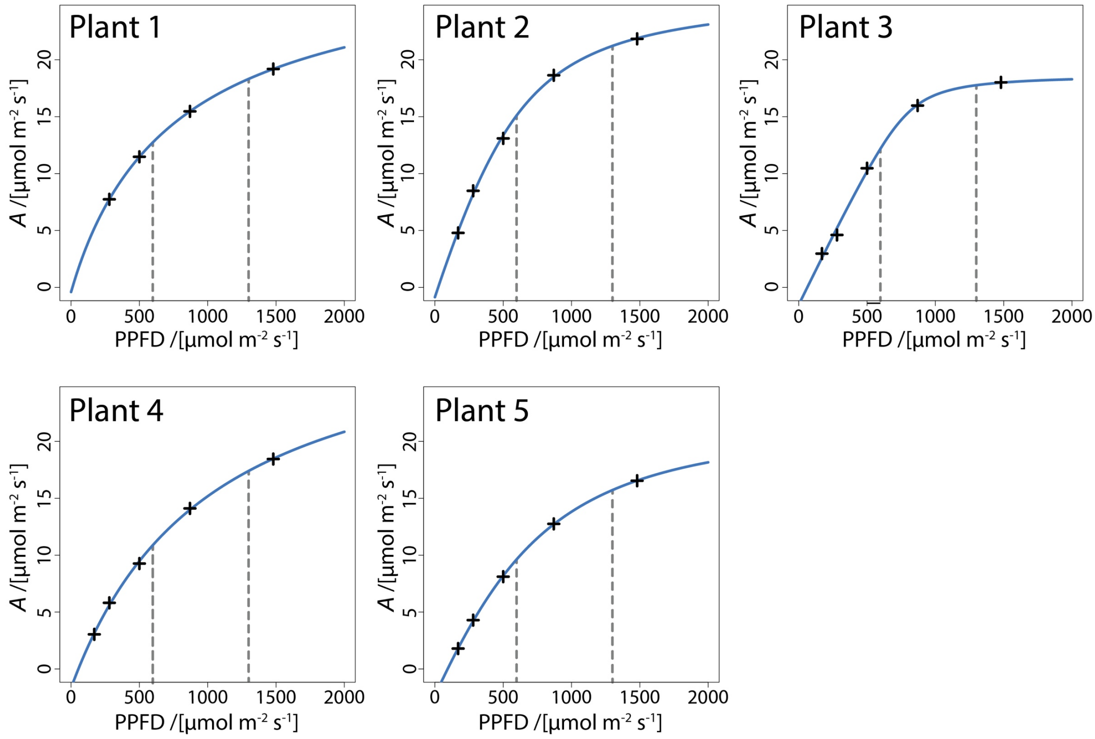

Figure S2 – Frequency distributions of 1/τ for the (a) Y/F DH population.

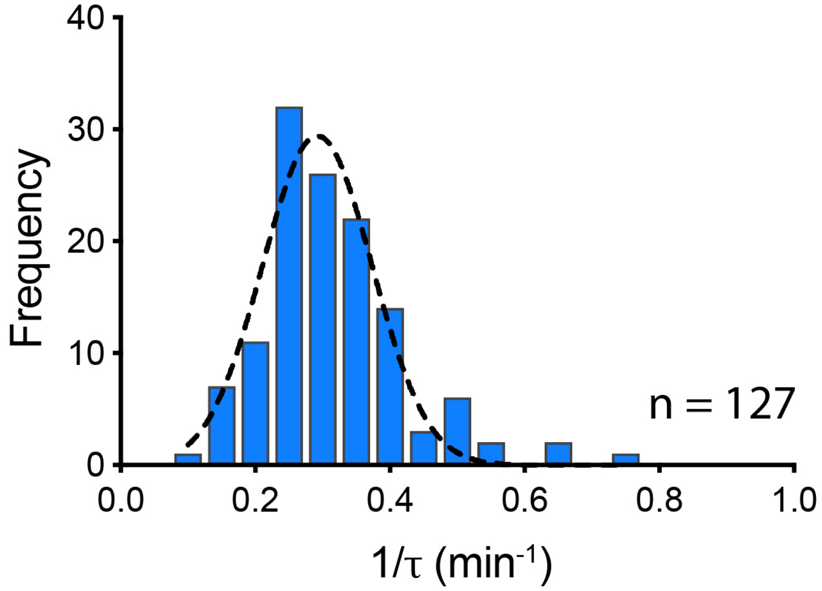

Figure S3 – Frequency distributions of steady-state (a) *A* and (b) g_s for the Y/F DH population.

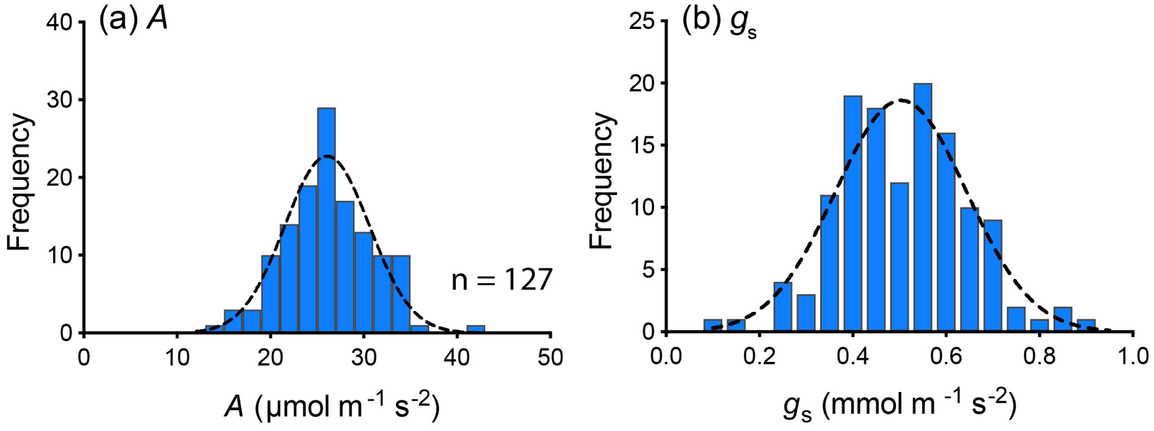

Figure S4 - Distribution of Rubisco activation rate (1/τ) across genotypes of the V/D DH population. Each bar represents a single genotype. Parental lines are highlighted. Colours are arbitrary.

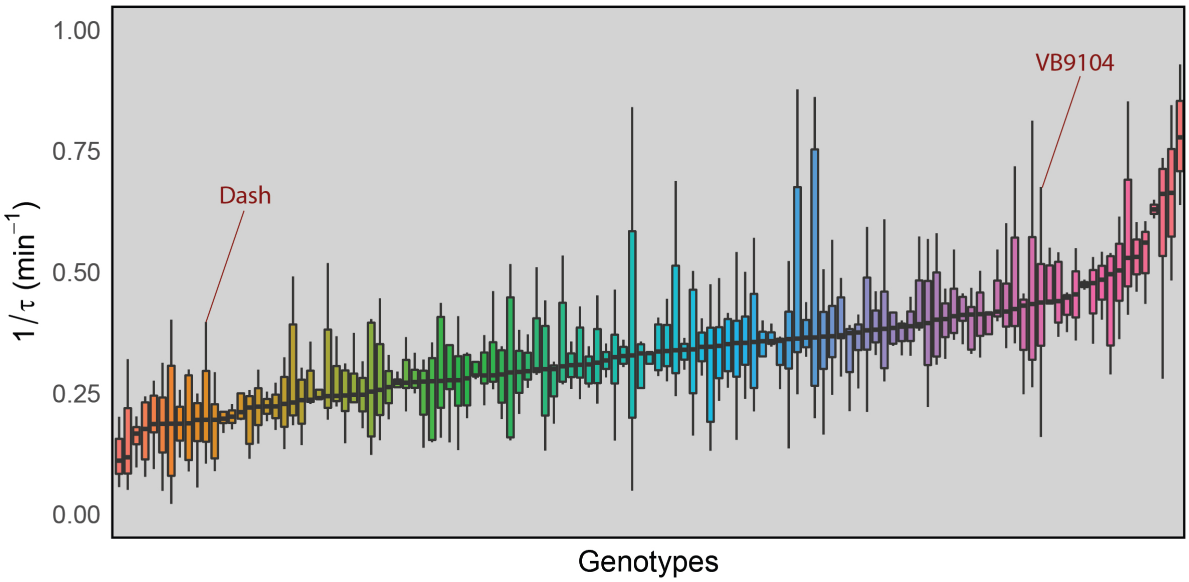

Figure S5 - Distribution of steady state (a) A and (b) g_s across genotypes of the VB9104/Dash population. Each bar represents a single genotype. Parental lines are highlighted. Note that colours are arbitrary but are consistent for genotypes in panels (a) and (b).

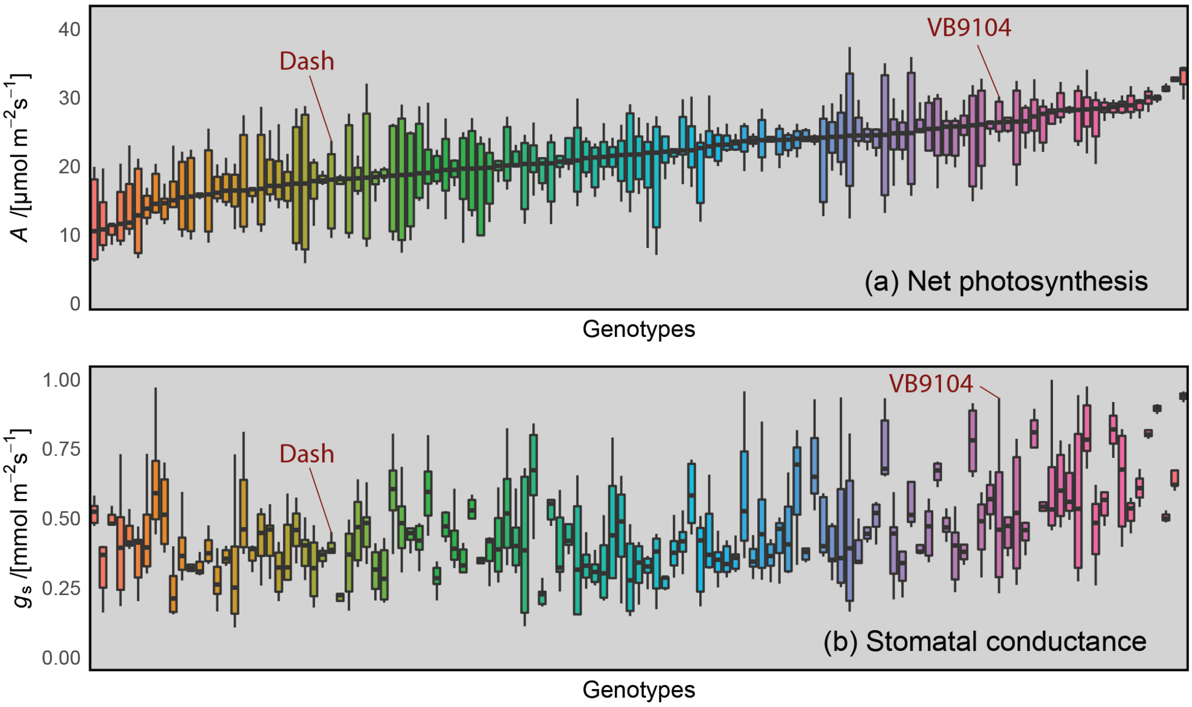

Supplement: plaa063_suppl_Supplementary_Figures [file plaa063_suppl_supplementary_figures.pdf]
